# Effects of Electronic Serious Games on Older Adults With Alzheimer’s Disease and Mild Cognitive Impairment: Systematic Review With Meta-Analysis of Randomized Controlled Trials

**DOI:** 10.2196/55785

**Published:** 2024-07-31

**Authors:** Xinyi Zuo, Yong Tang, Yifang Chen, Zhimiao Zhou

**Affiliations:** 1 Sociology Department School of Government Shenzhen University Shenzhen, Guangdong China; 2 Institution of Policy Studies Lingnan University Tuen Mun, Hong Kong SAR China (Hong Kong); 3 Shenzhen Senior High School Shenzhen China

**Keywords:** digital serious games, cognitive ability, daily behavioral capacity, mental health, depression, older adults with AD and MCI, AD, Alzheimer’s disease, MD, mild cognitive impairment, systematic review, meta-analysis

## Abstract

**Background:**

Serious games (SGs) are nonpharmacological interventions that are widely applied among older adults. To date, no evidence has been published regarding the effect of digital SGs on cognitive ability, daily behavioral capacity, or depression in older adults with Alzheimer’s disease (AD) and mild cognitive impairment (MCI).

**Objective:**

This study aimed to assess the effect of SGs on older adults with AD and MCI by summarizing and pooling the results of previous studies.

**Methods:**

This meta-analysis examined the effectiveness of digital SGs in improving cognitive ability, enhancing daily behavioral capacity, and alleviating depression in older adults with AD and MCI. We searched the following databases up to December 31, 2023, to identify relevant high-quality randomized controlled trials (RCTs): PubMed, Embase, Web of Science, Scopus, and Cochrane Library. Stata 15.1 and Review Manager 5.3 were used to screen the 14 studies, extract data, code the data, and perform meta-analysis. Mean differences and standardized mean differences (SMDs) with 95% CIs were used to calculate continuous variables. The Cochrane risk-of-bias assessment tool was used to evaluate the risk of bias. Eligibility criteria were developed in accordance with the Population, Intervention, Comparison, Outcomes, and Study Design framework: (1) population (older adults with AD and MCI), (2) intervention (digital SG intervention), (3) comparison (digital SG intervention vs routine health care), (4) outcomes (cognitive ability, daily behavioral capacity, and depression), and (5) study or research design (RCT). Sensitivity analysis was performed, and a funnel plot was constructed.

**Results:**

From January 2017 to December 2023, we enrolled 714 individuals across 14 RCTs, with 374 (52.4%) in the severe game group using digital SGs and 340 (47.6%) in the control group using traditional methods. The results of our meta-analysis indicated that using digital SGs in older adults with AD and MCI is more effective than traditional training methods in several key areas. Specifically, digital SG therapy signiﬁcantly increased cognitive ability, as found in the Mini-Mental State Examination (SMD 2.11, 95% CI 1.42-2.80; P<.001) and the Montreal Cognitive Assessment (SMD 2.75, 95% CI 1.98-3.51; P<.001), significantly increased daily behavioral capacity (SMD 0.53, 95% CI 0.06-0.99; P=.03), and significantly reduced depression (SMD –2.08, 95% CI –2.94 to –1.22; P<.001) in older adults with AD and MCI. No publication bias was detected based on the results of Begg and Egger tests.

**Conclusions:**

Digital SGs offer a viable and effective nonpharmacological approach for older adults with AD and MCI, yielding better results compared to traditional formats. However, caution is warranted in interpreting these findings due to limited RCTs, small sample sizes, and low-quality meta-analyzed evidence.

**Trial Registration:**

PROSPERO International Prospective Register of Systematic Reviews: CRDCRD42023486090; https://www.crd.york.ac.uk/PROSPERO/display_record.php?RecordID=486090

## Introduction

Alzheimer’s disease (AD) and mild cognitive impairment (MCI) are among the most common diseases affecting older adults. As the worldwide population is aging, the incidence of AD and MCI is gradually increasing, leading to major challenges for older adults. AD and MCI are being increasingly researched worldwide because of their adverse effects on cognitive ability, daily behavioral capacity, and depression among older adults. Currently, more than 55 million people worldwide have dementia [1]. Dementia is a progressive degenerative disease of the central nervous system that metastasizes through different clinical stages [2] and is characterized by abnormal behavior, declining cognitive function, and declining daily living ability [3]. Dementia affects multiple aspects of cognitive function, including complex attention, memory and learning, perceptual-motor skills, social cognition, and language [4]. AD is the most common form of dementia and may account for 60%-70% (38.77 million) of the 55 million cases of dementia [5]. MCI is an intermediate stage between dementia and normal aging [6]; clinicians and researchers consider the stage of MCI to be a “window” for potentially delaying the progression into dementia [7]. Moreover, the number of MCI cases among individuals above the age of 60 years is 5.82 million [8]. In 2019, the worldwide economic cost of dementia amounted to US $1.3 trillion, of which approximately 50% (US $0.65 trillion) involved care provided by informal caregivers (eg, family members and close friends), who provided an average of 5 hours of care and supervision per day [9]. China has the largest number of patients with dementia worldwide, which leads to a huge burden on the health care and public system [10]. Approximately 13.75 million (25%) of 55 million people with dementia are Chinese [11], and thus, this country ranks first in the world. The results of a recent national cross-sectional study showed that there are 16.07 million people aged 60 years and over in China who have dementia; 9.83 million of these individuals have AD [11]. Presently, there is no known cure for dementia; health care and medication can only slow the disease progression [12].

When older people get MCI and AD, they experience a range of painful experiences related to this particular stage of life, such as depression [7,13], decreased cognitive ability [7,14], and decreased daily living ability [15,16]. Sudden illness can cause patients to experience both physical and psychological pain [17]. A significant proportion of the population with MCI and AD reports higher levels of perceived [18]. Depression is characterized by threats that exceed an individual’s coping abilities, thus resulting in a feeling of being overwhelmed [19]. A growing body of evidence suggests that several important dimensions of life are frequently sacrificed when individuals get MCI and AD, and cognitive ability, daily behavioral capacity and depression are often neglected. Therefore, digital serious games (SGs) for older adults with MCI and AD may be crucial in the early intervention and prevention of these mental and physical disorders [20]. According to a previous report, even in worse cases, mental and physical disorders are risk factors for MCI and AD [21]. Patients with MCI and AD are completely dependent on caregivers, have markedly impaired memory function, are unable to take care of themselves in daily life, exhibit incontinence, and may even be completely unable to speak and have hemiplegia [22]. MCI and AD even cause an increase in the risk of mortality [23], which significantly damages people’s health and imposes a growing burden on individuals and the entire society. Therefore, it is highly emergent to develop nondrug therapies and use them in the field of social work for older adults. This may slow the progression of cognitive disability in individuals with MCI and AD to combat personal and socioeconomic problems, to address the alarming increase in the incidences of MCI and dementia worldwide, and to overcome the lack of effective drug treatments for these diseases.

There are many therapeutic methods for treating MCI and AD in older adults. Game therapy has been recommended as a nonpharmacological treatment method due to its limited side effects. According to recent meta-analyses [24-26], game therapy could increase cognitive ability [26] and daily performance ability [27,28] and alleviate depression [29,30] in older adults with MCI and AD. Game therapy refers to an intervention method in which games are used as a therapeutic medium. This therapy originated from a child psychoanalysis program developed by the famous psychologist Sigmund Freud. He observed that there is a relationship between early childhood development and children’s games. In the discussion of children’s control of emotions through games, Freud presented the rationale for game therapy [31]. After nearly a century of clinical development, the therapeutic function of games has been proven in practice [32,33]. SGs may take many forms, depending on their treatment, such as (1) cognitive training games, which are mainly used to improve or maintain users’ cognitive abilities (eg, memory, executive function, and memory) [34-37]; (2) sports games or video games, in which part of the game needs to include exercise [38,39]; (3) biofeedback games, which use electronic sensors connected to the participant’s body to receive information about their physical status (eg, an electrocardiograph sensor), with the aim of adjusting some bodily functions (eg, heart rate) [39-46]; and (4) cognitive behavioral therapy (CBT)–based games that provide computerized computer-based training. Due to the popularity of smart devices, handheld computers, personal computers, video game consoles, tablets, and smartphones, there is a wide variety of SGs [47,48]. Digital SGs, as defined in this paper, are SGs played through digital media. Digitally designed games help promote mental health [49], emotions [50], well-being [51], social function [51], and cognitive flexibility [52] compared to traditional cognitive training and exercise [53].

With respect to the development of game therapy theory, many studies have shown that game therapy is applicable for older adults [54]. The introduction of virtual reality technology has recently provided a new way to implement environmental enrichment in the context of clinical practice, which improves the physical health of patients with MCI and AD [55], treats chronic disease [56], increases cognitive ability [57] and daily behavioral capacity [50,52], alleviates depression [58], and increases self-esteem [59] and cohesion [60] by combining virtual immersion with cognitive stimulation. In conclusion, SG therapy is regarded as a promising treatment strategy for older adults with dementia and MCI in terms of increasing cognitive ability, improving daily behavioral capacity, and alleviating mental health issues. Game therapy is therefore increasingly used in gerontology, with the goal of providing psychosocial stabilization and support.

There are a few research gaps. First, there are inconsistent clinical results regarding the effects of SG therapy on cognitive performance, daily performance, and depression. According to Saragih et al [21] and Ning et al [60], SGs have been shown to improve cognitive function. However, Huang et al [61] and Kleschnitzki et al [62] reported that no significant changes are found in cognitive function or global status after virtual reality intervention. Wang et al [63] discovered that SGs have a positive impact on executive function. However, Abd-Alrazaq et al [52] found that there is no difference between adaptive SGs and nonadaptive SGs in improving executive function, although daily behavioral capacity is particularly related to executive function, such as scheduling appointments, making monthly payments, managing the household economy, shopping, or taking the bus. This means the effect of SGs in improving daily behavioral capacity is yet to be explored. Bai et al [50] compared SGs with intervention-free practices and found that game-based interventions lead to clinically and statistically significant improvements in depression severity. Bojan et al [64] discovered that SGs can cause a significant increase in positive emotions and a decrease in negative emotions [11,65]. However, according to Fitzgerald et al [66], whether SGs can improve depression in some cases is worth exploring. Therefore, there are inconsistent clinical results regarding the effects of SG therapy on cognitive performance, daily performance, and depression. A more comprehensive review is needed to systematically evaluate the effects of SGs on the physical and mental health of older adults with AD and MCI. Second, the previous literature only focused on SGs for a type of disease for older adults. Manca et al [67] observed the positive impact of SGs with humanoid robots on older adults with MCI. Pacheco et al [68] found that exergames improve balance and mobility in older adults to keep performing balance exercises. Yu and Chan [69] discovered video game training can effectively improve the cognition of older adults. This study investigated the effects of SGs on the cognitive ability, daily behavioral capacity, and depression of older adults with MCI and AD simultaneously. Third, it is important to investigate not only the effects of SGs but also the variables that may influence their effectiveness, including the duration of treatment and the target population. In this research, we used weeks and continental plates (within and outside Europe) to divide the subgroup analysis, which was not available in previous studies. In view of this, we conducted a systematic review and meta-analysis to comprehensively evaluate the effect of digital SGs on cognitive ability, daily behavioral capacity, and depression of older adults (≥60 years old) with MCI and AD.

## Methods

### Data Sources and Search Strategy

This review was conducted in strict accordance with PRISMA (Preferred Reporting Items for Systematic Reviews and Meta-Analyses) guidelines [70]; also see Multimedia Appendices 1 and 2 [71]. We registered the study in PROSPERO (Prospective Register of Systematic Reviews; CRD42023486090). We used the PICOS (Population, Intervention, Comparison, Outcomes, and Study Design) framework [72] to structure study inclusion and exclusion criteria (Table 1). Our population of interest had at least 1 indicator indicating cognitive ability, behavioral ability, or emotion-related ability, and their age was ≥60 years. We sought studies describing interventions with digital SGs implemented among older adults with cognitive impairment in an experimental group (severe game group [SGG]), including exergames or video games, cognitive training games, computerized CBT-based games, and biofeedback games. There were no restrictions regarding the time of intervention. For comparison, we had a control group (CG) that received a different intervention, such as routine health care, traditional Tai Chi, or music. Our outcomes of interest included cognitive ability, daily behavioral capacity, and depression in older adults with cognitive impairment. We excluded studies that presented reviews, non–randomized controlled trials (RCTs), and papers for which the full text was unavailable. All included studies were published in Chinese or English. Studies in which the results were interpreted from the perspective of older adults with cognitive impairment were deemed eligible.

**Table 1:** Inclusion and exclusion criteria.

Element	Inclusion criteria	Exclusion criteria
Population	At least 1 indicator representing cognitive ability, behavioral ability, or emotion-related ability Age≥60 years	N/A^a^
Intervention	Digital SGs^b^ (eg, exergames, video games, cognitive training games, computerized CBT^c^-based games, biofeedback games)	N/A
Comparison	Different interventions (eg, routine health care, traditional Tai Chi, music)	N/A
Outcomes	Cognitive ability Daily behavioral capacity Depression	N/A
Study design	RCT^d^	Reviews Non-RCTs Papers with full text unavailable
Language	Chinese or English papers	Not Chinese or English papers

^a^N/A: not applicable.

^b^SG: serious game.

^c^CBT: cognitive behavioral therapy.

^d^RCT: randomized controlled trial.

### Search Strategy

The following databases were searched to identify the relevant literature: PubMed, Embase, Web of Science, Scopus, and Cochrane Library. The following search terms were used for the search: (“Cognitive Ability” OR “Daily Behavior Ability” OR “Mental Health” OR “Depression”) AND (“Serious Games Based” OR “Serious Games” OR “Web-Based or Mobile”) AND (“Old Adults in Cognitive Impairment” OR “Old Adults” OR “Older People”) AND (“A pilot study” OR “Randomized Controlled Trial” OR “RCT”). Detailed search strategies for each database are given in Multimedia Appendix 3. The keywords were used to search for papers published from January 1, 2017, to December 31, 2023. The snowball method was adopted to search the reference lists of the included studies. Additionally, the reference lists of the included studies were manually searched to identify eligible papers. Unpublished academic literature was not considered eligible. The reference lists and relevant reviews were then screened to identify any pertinent studies. To identify additional relevant publications, we also retrieved gray literature [73].

### Data Extraction

Four researchers (authors XZ, YT, YC, and ZZ) screened all the literature for inclusion. After removing duplicates using EndNote X9, all studies were independently screened based on titles and abstracts. Next, we carefully read the full texts of the remaining papers in accordance with the inclusion and exclusion criteria. Finally, data were extracted from the included studies. The co-first author (XZ) used the modified version of the data extraction table in the *Systematic Review Manual of Cochrane Interventions* to extract data. A modified version of the intervention description and replication was using following the template for intervention description and replication (TIDieR) list and guidelines [74] to extract data of the intervention, design, and delivery features, as defined in the introduction. First, data were extracted by the co-first author (XZ). Next, the second author (ZZ) checked the accuracy of the process. Finally, the co-first author (YC), through thematic analysis [75], extracted the following data from the list of 14 studies [34-47]:

- Study characteristics: name of author, publication year, country, publication type, and study design
- Subject characteristics: sample size, participant mean age, participant age, Mini-Mental State Examination (MMSE) score, participant health conditions, and population group
- Intervention details: setting, intervention type, delivery mode design (how), content (ie, procedures, materials, process, activities [what]), and delivery details (ie, intervention delivery format, intervention duration, session length, session frequency, intensity)
- Study quality: Jadad score
- Main outcomes: reported results, conclusion, measurement, and follow-up

### Risk-of-Bias Assessment and Quality Assessment

Using the Cochrane Collaboration risk-of-bias tool [76], we independently assessed the risk of bias. Additionally, we assessed the quality of the 14 studies using the Jadad scale [77]. The risk-of-bias tool assesses 7 domains: (1) generation of a random sequence, (2) allocation concealment, (3) subject and experimenter blinding, (4) outcome assessor blinding, (5) resulting data integration, (6) selective reports, and (7) other bias risk. Each study was categorized as having a low, high, or unclear risk of bias. We also performed Begg and Egger tests to evaluate the degree of publication bias [78].

### Statistical Analysis

Stata 15.1 and Review Manager 5.3 were used to conduct data analysis. Forest plots were constructed to intuitively illustrate the results. In the included literature, the outcomes were measured as continuous variables, and the same indicator was assessed with different tools. These indicators were presented as standard mean differences (SMDs). α=.05 was considered statistically significant. Heterogeneity was assessed with *I*^2^ statistics, which were classified as high (>75%), moderate (50%-75%), or low (<50%) [79]. In cases of high heterogeneity, sensitivity analysis was performed using the leave-1-out method to identify the sources of heterogeneity. In the analysis, numerous weeks were compared as subpoints to check the results. The Begg test [80] and Egger test [81] were used to assess publication bias. P<.05 indicated significant results. When a meta-analysis included at least 10 studies, publication bias was evaluated with a funnel plot [82,83]. In this study, 95% CI and the SMD were examined [84]. Overall, P<.05 indicated statistically significant effects. Subgroup analyses were performed based on the country of intervention (outside Europe or not) and the length of intervention (weeks).

## Results

### Study Selection

**Figure 1:**
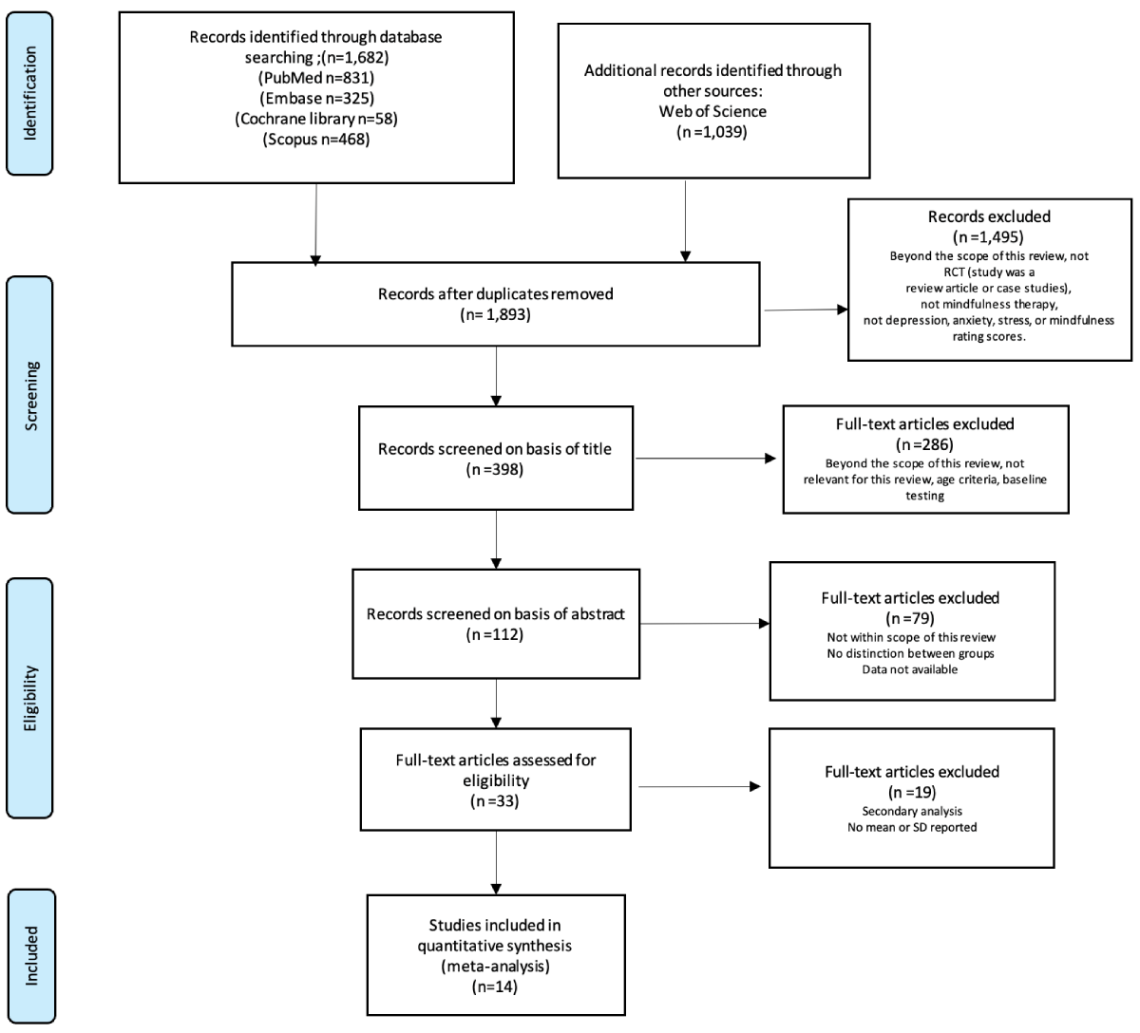
Study selection flowchart based on PRISMA guidelines, 2020. PRISMA: Preferred Reporting Items for Systematic Reviews and Meta-Analyses.

Figure 1 shows the study selection process. After searching 13 databases, 4260 relevant records were identified. For duplicate removal, we imported all the studies into EndNote X8 [85]. After removing 1893 (44.4%) duplicates and eliminating 2352 (55.2%) papers via a strict screening process, 14 (0.3%) RCTs [34-47] from before December 2023 involving 714 participants were finally included. Studies were excluded because they did not report SDs [26,86-88], were review papers [89,90], or were not within the scope of this meta-analysis [91-94]. All the included studies reported that digital SGs have a positive impact on cognitive ability, daily behavioral capacity, and depression. The primary outcomes of interest were an increase in cognitive ability scores (assessed with the MMSE and the MoCA), an increase in daily behavioral capacity indicators (assessed with the Barthel Index, the Katz Index, the Lawton and Brody Index, the Korean version of the Modified Barthel Index [K-MBI], and the Activities of Daily Living [ADL] scale), and a reduction in depression (assessed with the Goldberg Anxiety and Depression Scale [EADG], the 15-item Geriatric Depression Scale [EDG-15], and the Cornell Scale for Depression in Dementia [CSDD]).

### Study Characteristics

Table 2 and Multimedia Appendix 4 [34-47] show the overall characteristics of the included studies. All 14 studies were published before 2023. The sample sizes ranged from 17 to 112, and 714 older adults aged >60 years were enrolled in the included studies, including 340 (47.6%) subjects in the SGG and 374 (52.4%) subjects in the CG. All participants were older adults with cognitive impairment, but they had not been diagnosed with psychiatric disorders. All interventions were based on digital SGs, and their durations ranged from 4 weeks to 12 weeks. The interventions lasted from 45 minutes to 5 hours each week. Both group training and individual training methods were used [34-47]. All 14 studies [34-47] were subdivided into digital SG training methods (exergames or video games, cognitive training games, computerized CBT games, and biofeedback games).

**Table 2:** Characteristics and findings of the included studies (N=14)^a^.

Study	Location	Sample size: SGG^b^ and CG^c^, n/N (%)	Age (years): SGG and CG, range (average)/mean (SD)	Disease	Time frame (weeks)	Reported results and conclusion
Yang et al [34]	Republic of Korea	SGG: 10/20 (50) CG: 10/20 (50)	SGG: 64-78 (71.1) CG: 61-78 (69.9)	Dementia	4	Result: Postintervention, the K-MMSE^d^ score of the SGG was higher than that of the CG. Conclusion: Computer-based CBT^e^ training might have beneficial effects on general cognitive function in the early stage of AD^f^.
Kwan et al [35]	Hong Kong, China	SGG: 16/33 (48) CG: 17/33 (52)	SGG: (average): 70.5 CG: (average): 71	Dementia	12	Result: Postintervention, the MoCA^g^ score of the SGG was higher than that of the CG. Conclusion: The mobile health (mHealth) intervention is feasible for implementation in older people with cognitive impairment and is effective at enhancing cognitive function.
Lim et al [36]	Republic of Korea	SGG: 12/24 (50) CG: 12/24 (50)	SGG: 69-81 (75.42) CG: 65-90 (73.33)	Dementia	4	Result: Postintervention, the K-MMSE, K-MoCA^h^, and DQoL^i^ scores of the SGG were higher than those of the CG. Conclusion: The research group showed significant improvement in cognitive function after training. Family SGs^j^ are believed to help improve cognitive function.
Lee et al [37]	Republic of Korea	SGG: 10/20 (50) CG: 10/20 (50)	SGG: 65-83 (74.5) CG: 65-82 (74)	MCI, AD, dementia	3	Result: Postintervention, the K-MMSE and ADL^k^ scores of the SGG were higher than those of the CG. Conclusion: Through this preliminary study, we verified that the newly developed computerized cognitive rehabilitation program is effective in improving cognitive function.
Yang [38]	Fujian, China	SGG: 44/88 (50) CG: 44/88 (50)	SGG: 62-83 (71.89) CG: 61-82 (71.63)	Dementia	12	Result: Postintervention, the MMSE^l^ and ADL scores of the SGG were higher than those of the CG. Conclusion: Interactive games can improve the cognitive function and daily living ability of patients with senile dementia.
Van Santen et al [39]	Netherlands	SGG: 73/112 (65) CG: 39/112 (35)	SGG: 73-85 (79) CG: 72-86 (79)	Dementia	8	Result: Postintervention, the MMSE score of the SGG was higher than that of the CG. Conclusion: Sports games can improve the cognitive function of patients with senile dementia.
Zheng et al [40]	Zhejiang, China	SGG: 18/38 (47) CG: 20/38 (53)	SGG: 75-87 (81.74) CG: 78-89 (84.26)	Dementia	8	Result: Postintervention, compared to the CG, the MMSE score of the SGG was significantly higher and the CSDD^m^ score was significantly lower. Conclusion: Somatic interactive game interventions can improve memory and language function and reduce depression in older patients with dementia, but the impact on cognitive function and ADL needs to be explored.
Wu et al [41]	Shandong, China	SGG: 47/94 (50) CG: 47/94 (50)	SGG: 61-80 (71.74) CG: 60-80 (72.65)	Dementia	12	Result: Postintervention, the MoCA score of the SGG was higher than that of the CG. Conclusion: Sensory stimulation combined with sensory interaction games can improve cognitive function.
Savulich et al [42]	United Kingdom	SGG: 21/42 (50) CG: 21/42 (50)	SGG: 67-82 (75.2) CG: 68-85 (76.9)	Dementia	4	Result: Postintervention, the MMSE score of the SGG was higher than that of the CG. Conclusion: Gamification maximizes engagement with cognitive training by increasing motivation and could complement pharmacological treatments for amnestic MCI^n^ and mild AD.
Swinnen et al [43]	Belgium	SGG: 23/45 (51) CG: 22/45 (49)	SGG: 79-90 (84.7) CG: 78-91 (85.3)	Dementia	8	Result: Postintervention, compared to the CG, the MMSE, MoCA, and DQoL scores of the SG were higher and the CSDD score was significantly lower. Conclusion: Individually adapted exergame training improves lower extremity functioning, cognitive function, and depression in long-term-care facilities.
Liu et al [44]	Taipei, China	SGG: 16/33 (48) CG: 17/33 (52)	SGG: 68-80 (74.6) CG: 66-79 (73.4)	Dementia	12	Result: Postintervention, the MoCA score of the SGG was higher than that of the CG. Conclusion: Exergaming-based Tai Chi is comparable to traditional Tai Chi for enhancement of cognitive function.
Thapa et al [45]	Republic of Korea	SGG: 34/68 (50) CG: 34/68 (50)	SGG: 67-78 (72.6) CG: 67-78 (72.7)	MCI, dementia	8	Result: Postintervention, the MMSE score of the SGG was higher than that of the CG. Conclusion: Encouraging patients to perform virtual reality– and game-based training may be beneficial to prevent cognitive decline.
Oliveira et al [46]	Portugal	SGG: 10/17 (59) CG: 7/17 (41)	SGG: 77-88 (82.6) CG: 77-90 (84.14)	Dementia	5	Result: Postintervention, the MMSE and ADL scores of the SGG were higher than those of the CG. Conclusion: This approach is effective for neurocognitive stimulation in older adults with dementia, contributing to maintaining cognitive function in AD.
Jahouh et al [47]	Spain	SGG: 40/80 (50) CG: 40/80 (50)	SGG: 85.05 (8.63) CG: 83.25 (8.78)	MCI	8	Result: Postintervention, the MMSE and ADL scores of the SGG were significantly higher and the EGD-15^o^ and EADG^p^ scores were significantly lower than those of the CG. Conclusion: The Wii video console has a positive influence on older people by increasing cognitive ability, enhancing the level of ADL, and improving the psychological status.

^a^Publication type: journal paper; study design: randomized controlled trial (RCT); study population: older adults; setting: clinical.

^b^SGG: serious game group.

^c^CG: control group.

^d^K-MMSE: Korean version of the Mini-Mental State Examination.

^e^CBT: cognitive behavioral therapy.

^f^AD: Alzheimer’s disease.

^g^MoCA: Montreal Cognitive Assessment.

^h^K-MoCA: Korean version of the Montreal Cognitive Assessment.

^i^DQoL: Dementia Quality of Life.

^j^SG: serious game.

^k^ADL: activities of daily living.

^l^MMSE: Mini-Mental State Examination.

^m^CSDD: Cornell Scale for Depression in Dementia.

^n^MCI: mild cognitive impairment.

^o^EGD-15: 15-item Geriatric Depression Scale.

^p^EADG: Goldberg Anxiety and Depression Scale.

### Risk of Bias and Quality Assessment

**Figure 2:**
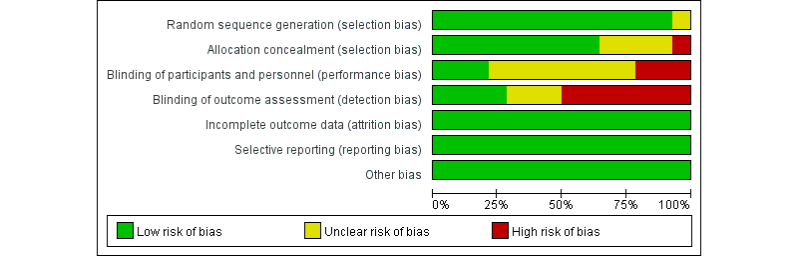
Risk-of-bias graph.

**Figure 3:**
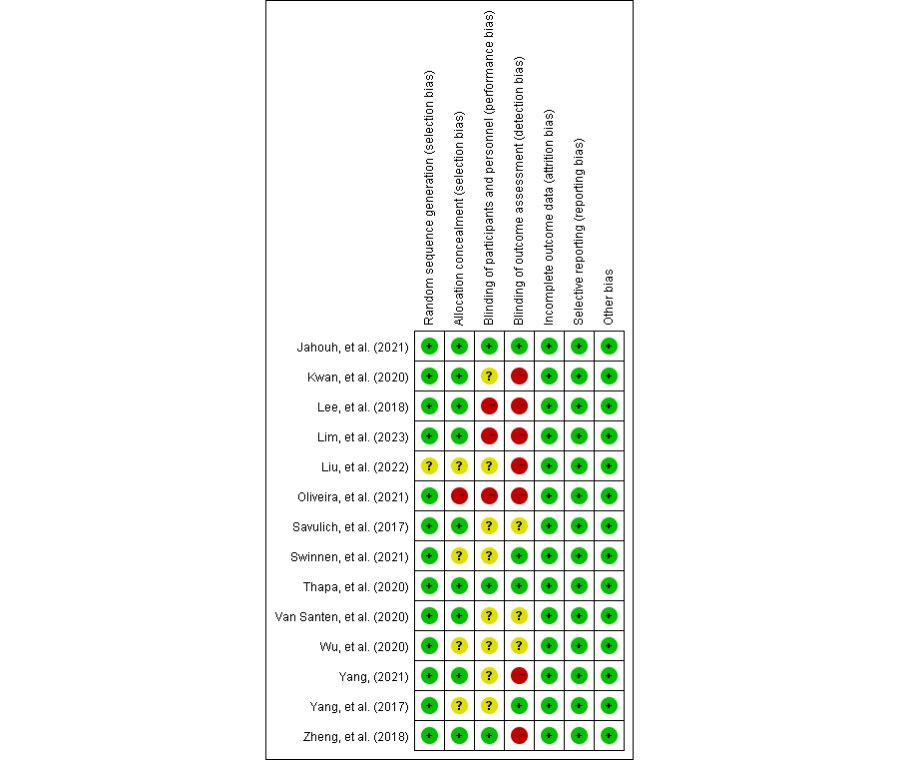
Risk-of-bias summary of the 14 studies included.

Figures 2 and 3 show the results of the risk-of-bias assessment. All 14 papers [34-47] described the randomization methods in detail. The blinding method was detailed in 7 (50%) studies: 4 (57%) of these studies were double-blind trials [40,44,45,47], while 3 (43%) were single-blind trials [36,37,46]. The average Jadad score across all included studies was 4.7, indicating fair-to-mild quality.

### Meta-Analyses

#### Cognitive Ability Indicators

**Figure 4:**
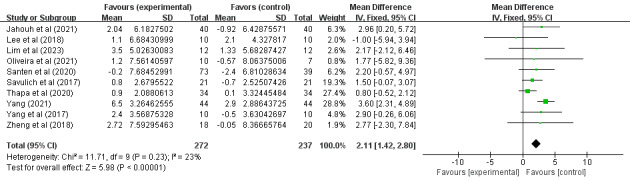
Forest plot for MMSE. MMSE: Mini-Mental State Examination.

**Figure 5:**
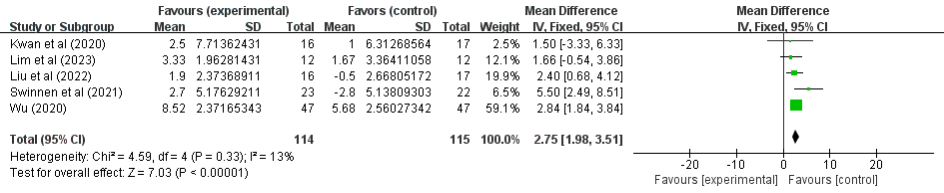
Forest plot for MoCA. MoCA: Montreal Cognitive Assessment.

Of the 14 studies, 10 (71%) [34,36-40,42,45-47] involving 409 (57.3%) older adults (n=272, 66.5%, in the SGG and n=237, 33.5%, in the CG) assessed the effects of SGs on older adults’ cognitive ability based on MMSE scores. Because different evaluation tools were used, the SMD was considered the pooled effect size measure. The pooled results revealed a low degree of heterogeneity among the studies (P=.23, *I*^2^=23%), necessitating the use of a random effects model for the meta-analysis. Figure 4 demonstrates that cognitive ability scores in digital SGs significantly increased between the 2 groups under investigation (SMD 2.11, 95% CI 1.42-2.80; P<.001). Of the 14 studies, 5 (36%) [35,36,41,43,44] involving 229 (32.1%) older adults (n=114, 49.8%, in the SGG and n=115, 50.2%, in the CG) assessed the effects of SGs on older adults’ cognitive ability based on MoCA scores. Because different evaluation tools were used, the SMD was considered the pooled effect size measure. The pooled results revealed a low degree of heterogeneity among the studies (P=.33, *I*^2^=13%), necessitating the use of a random effects model for the meta-analysis. Figure 5 demonstrates that cognitive ability scores in digital SGs significantly increased between the 2 groups under investigation (SMD 2.75, 95% CI 1.98-3.51; P<.001).

#### Daily Behavioral Capacity Indicators

**Figure 6:**
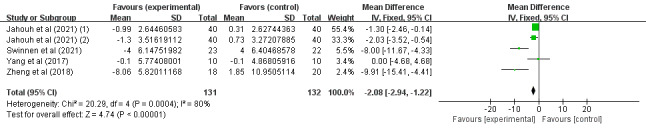
Forest plot for daily behavioral capacity indicators.

Of the 14 studies, 6 (43%) [37,38,40,43,46,47] involving 448 (62.7%) older adults (n=225, 50.2%, in the SGG and n=223, 49.8%, in the CG) assessed the effects of SGs on older adults’ daily behavioral capacity based on the Barthel Index, the Katz Index, the Lawton and Brody Index, the K-MBI, and the ADL scale. Because different evaluation tools were used, the SMD was considered the pooled effect size measure. The pooled results revealed significant heterogeneity among the studies (P=.03, *I*^2^=91%), necessitating the use of a random effects model for the meta-analysis. Figure 6 demonstrates that daily behavioral capacity scores in digital SGs significantly increased between the 2 groups under investigation (SMD 0.53, 95% CI 0.06-0.99; P=.03).

#### Depression Indicators

**Figure 7:**
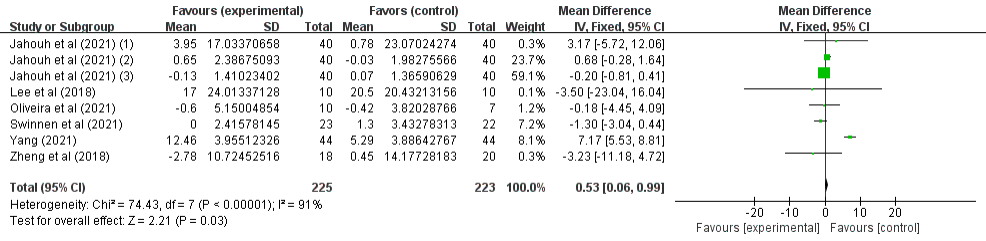
Forest plot for depression indicators.

Of the 14 studies, 4 (29%) [34,40,43,47] involving 292 (40.9%) older adults (n=145, 49.7%, in the SGG and n=147, 50.3%, in the CG) assessed the effects of SGs on older adults’ depression based on the EADG, the EDG-15, the Geriatric Depression Scale (GDS), and the CSDD. Because different evaluation tools were used, the SMD was considered the pooled effect size measure. The pooled results revealed a low degree of heterogeneity among the studies (P<.001, *I*^2^=80%), necessitating the use of a random effects model for the meta-analysis. Figure 7 demonstrates that cognitive ability scores in digital SGs significantly increased between the 2 groups under investigation (SMD –2.08, 95% CI –2.94 to 1.22; P<.001).

In conclusion, the included studies reported scores on the MMSE, MoCA, the Barthel Index, the Katz Index, the Lawton and Brody Index, the K-MBI, the ADL scale, the EADG, the EDG-15, and the CSDD. A total of 3 outcomes were evaluated: cognitive ability, daily behavioral capacity, and depression. The SGG had significantly greater total MMSE scores than the CG (SMD 2.11, 95% CI 1.42-2.80; P=.23, *I*^2^=23%), as shown in Figure 4. The SGG had significantly greater total MoCA scores than the CG (SMD 2.75, 95% CI 1.98-3.51; P=.33, *I*^2^=13%), as shown in Figure 5. In addition, a reduction in daily behavioral capacity indicators was observed based on the Barthel Index, the Katz Index, the Lawton and Brody Index, the K-MBI, and the ADL scale. The SG led to increased daily behavioral capacity scores in the SGG (SMD 0.53, 95% CI 0.06-0.99; P=.03, *I*^2^=91%), as shown in Figure 6, compared to the CG. The SGG also showed a decrease in depression based on the EADG, the EDG-15, and the CSDD (SMD –2.08, 95% CI –2.94 to –1.25; P<.001, *I*^2^=80%), as shown in Figure 7.

### Subgroup Analyses

**Figure 8:**
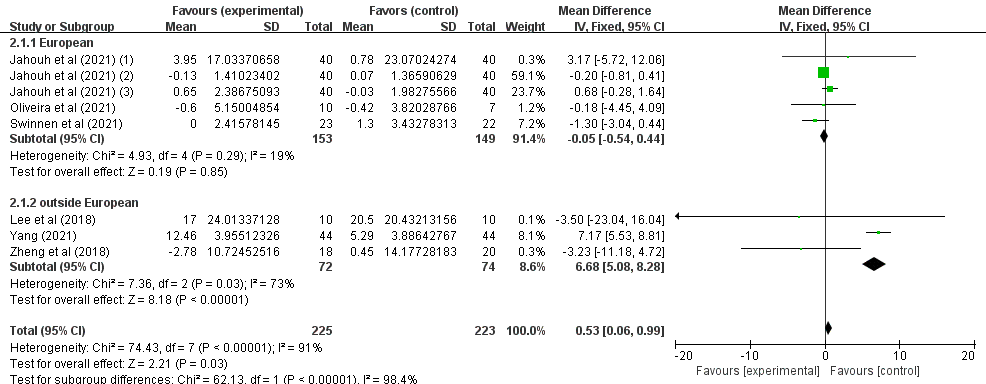
Forest graph showing subgroup analysis of daily behavioral capacity based on the country of the intervention.

**Figure 9:**
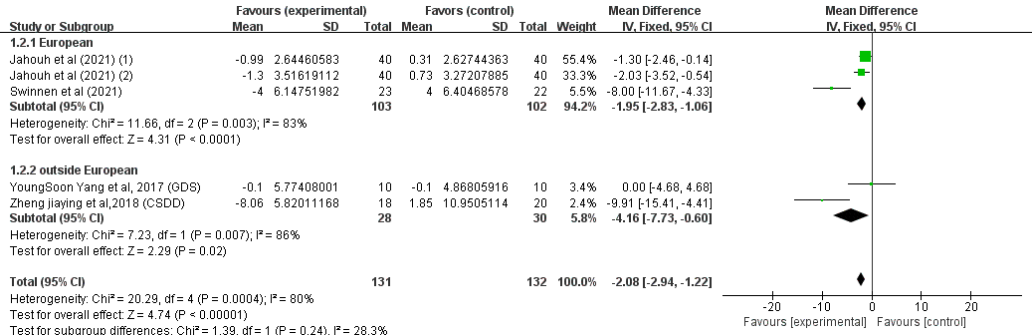
Forest graph showing subgroup analysis of depression based on the country of the intervention. CSDD: Cornell Scale for Depression in Dementia; GDS: Geriatric Depression Scale.

**Figure 10:**
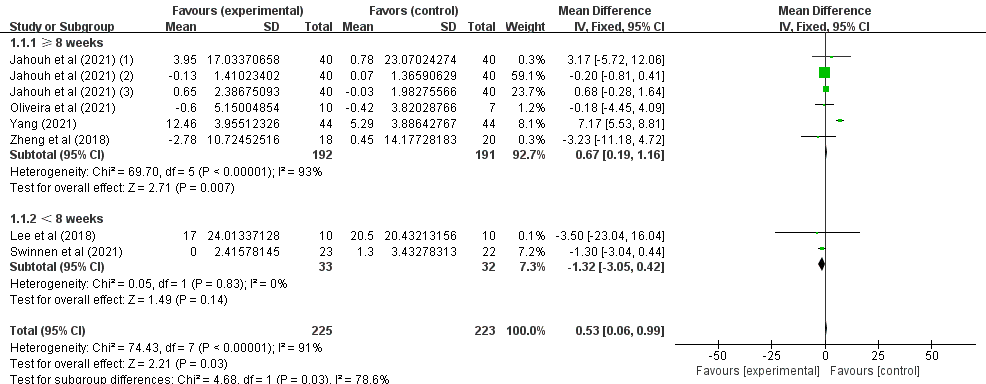
Forest graph showing subgroup analysis of daily behavioral capacity based on the duration of the intervention.

**Figure 11:**
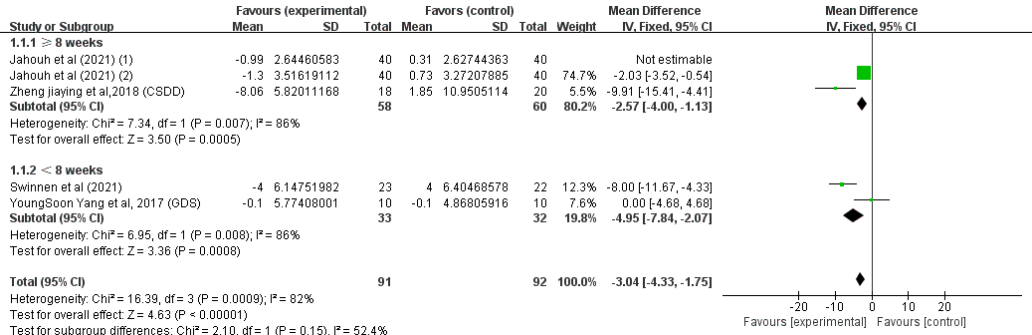
Forest graph showing subgroup analysis of depression based on the duration of the intervention. GDS: Geriatric Depression Scale.

Subgroup analyses of daily behavioral capacity and depression scores were performed based on the country of intervention (outside or within Europe) and the duration of intervention (weeks). For daily behavioral capacity, significant differences were found in the SMD between the 2 subgroups: within Europe (P=.85) [43,46,47] and outside Europe (P<.001) [37,38,40]. SGs had a significant effect on the outcome in the outside-Europe group (SMD 6.68, 95% CI 5.08-8.28; P<.001), as shown in Figure 8. For depression, significant differences were found in the SMD between the 2 subgroups: within Europe (P<.001) [43,47] and outside Europe (P=.02) [34,40]. SGs had a significant effect on the outcome in the within-Europe group (SMD –2.08, 95% CI –2.94 to –1.22; P<.001), as shown in Figure 9. Of the 6 (43%) studies, 4 (67%) [38,40,46,47] reported a pooled effect of an intervention duration of ≥8 weeks (SMD 0.67, 95% CI 0.19-1.16; P=.007). For the remaining 2 (33%) studies [37,43], the pooled effect within the intervention period was as follows: SMD −1.32, 95% CI −3.05 to −0.42; P=.14. The effects of SGs on daily behavioral capacity during the 2 periods of intervention were significantly different from those in the CG (P<.001), as shown in Figure 10. For depression, no significant differences were found in the SMD between the 2 groups after >8 weeks (P=.005) [40,47] or <8 weeks (P=.008) [34,43], as shown in Figure 11. Among the 4 (29%) studies on depression indicators [34,40,43,47], 2 (50%) [40,47] had a combined effect in an intervention duration of ≥8 weeks (SMD −2.57, 95% CI −4.00 to −1.13; P=.005). For 2 (50%) studies [34,43] in which the intervention duration was <8 weeks, the combined effect within the intervention period was as follows: SMD −4.95, 95% CI −7.84 to −2.07; P=.008. An intervention period of 8 weeks or more had a significant effect on reducing cognitive impairment in older adults (Figure 11). Compared to with the CG, the effects of SGs on depression during the 2 periods of intervention were significantly different, and a significant difference between the 2 groups was found (P<.001), as shown in Figure 11.

### Sensitivity Analysis

In the sensitivity analysis (Table 3), we excluded the study by Yang et al [38] and observed a significant change in heterogeneity from 91% to 0%. We hypothesized that the outcome of daily behavioral capacity indicators may have been the possible source of heterogeneity in this study. Additional possible reasons included the inclusion of patients, specific treatment modalities, and inconsistencies in clinical indicators between domestic and foreign countries. The patients included and specific treatment modalities, as well as the clinical indicators applied, in this study were as follows: Yang et al [38], Fujian (China), age 61-83 years; older adults with cognitive impairment, participated in somatosensory interactive games, and completed the MMSE and the ADL scale. All these different factors were possible sources of heterogeneity.

**Table 3:** Sensitivity analysis for daily behavioral capacity indicators after excluding a few studies.

Study excluded	Tool used	Heterogeneity analyses		
		*χ*² (*df*)	P value	*I*^2^ (%)
Jahouh et al [47]	Barthel Index	74.09 (6)	<.001	92
Jahouh et al [47]	Katz Index	61.17 (6)	<.001	90
Jahouh et al [47]	Lawton and Brody Index	74.02 (6)	<.001	92
Lee et al [37]	K-MBI^a^	74.01 (6)	<.001	92
Oliveira et al [46]	IADL^b^	74.07 (6)	<.001	92
Swinnen et al [43]	ADL^c^	69.67 (6)	<.001	91
Yang et al [34]	ADL	5.18 (6)	.52	0
Zheng et al [40]	ADL	73.31 (6)	<.001	92

^a^K-MBI: Korean version of the Modified Barthel Index.

^b^IADL: Instrumental Activities of Daily Living.

^c^ADL: activities of daily living.

### Publication Bias

**Figure 12:**
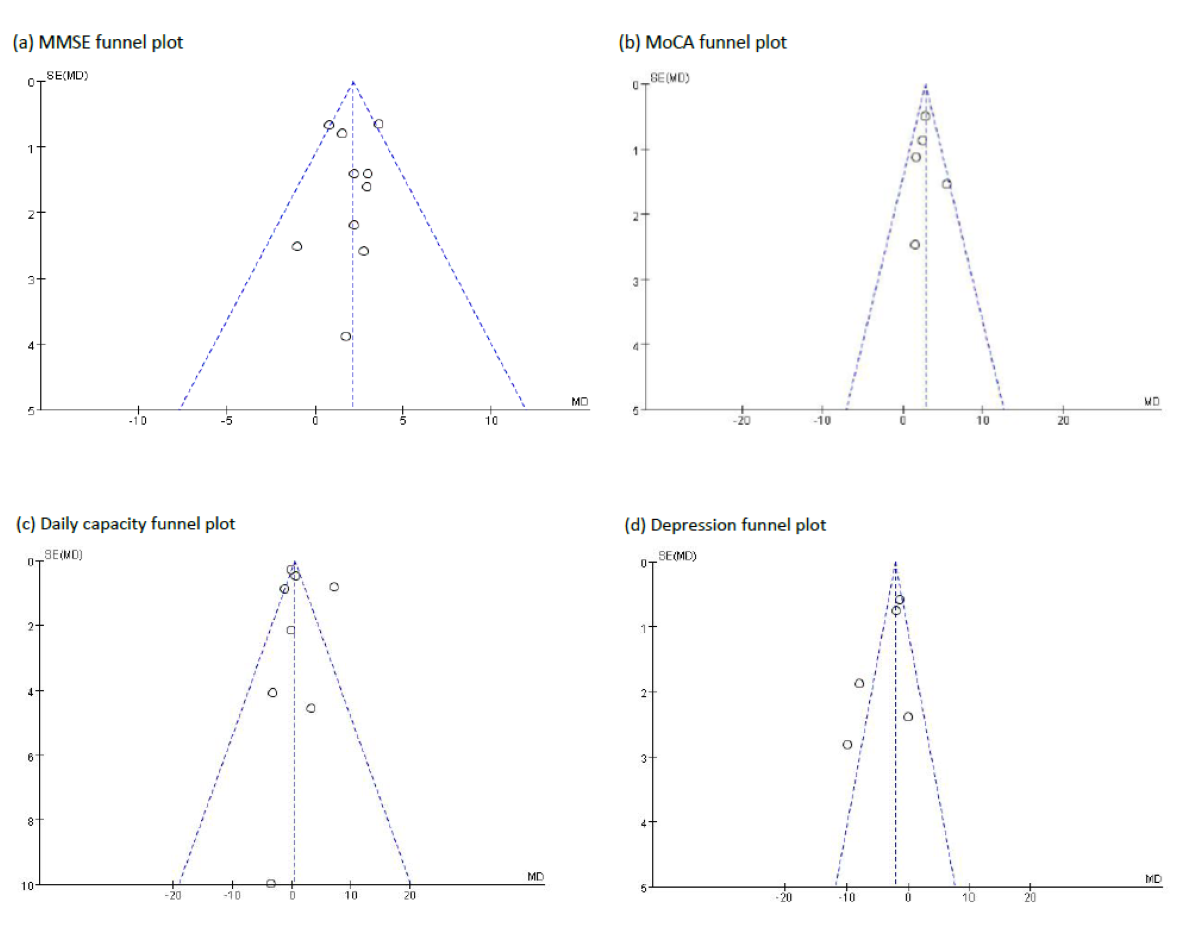
Funnel plots for (a) MMSE, (b) MoCA, (c) daily behavioral capacity indicators, and (d) depression indicators. MMSE: Mini-Mental State Examination; MoCA: Montreal Cognitive Assessment.

We found that the funnel plots for the MMSE (Figure 12a), MoCA (Figure 12b), daily behavioral capacity indicators (Figure 12c), and depression indicators (Figure 12d) were all symmetrical, indicating the absence of publication bias. P>.05 indicated the absence of obvious publication bias. The Begg test (P=.03) and Egger regression (P=.026) also indicated a lack of publication bias.

## Discussion

### Principal Findings

This study systematically evaluated the efficacy of training older adults with cognitive impairment using digital SGs, drawing upon data from 14 studies with a total of 714 participants. Our findings revealed that digital SGs significantly alleviate depression and yield large improvements in cognitive ability and daily behavioral capacity. This suggests that SGs offer a highly effective alternative for older adults with cognitive impairment. Due to statistical heterogeneity, these results should be interpreted with caution. In this study, sensitivity analysis was used to explore the major sources of statistical heterogeneity to determine the impact of a single study on overall risk. The type of intervention (various digital SGs), sample size (range 17-112), intervention duration (range 4-12 weeks), weekly intervention time (range 45-300 minutes/week), type of CG (traditional Taijiquan group, routine health care group, music group), cultural background, measurement tools, or other confounding factors may have caused heterogeneity. To the best of our knowledge, this is the first meta-analysis and systematic review evaluating the effectiveness of the 3 outcome indicators of serious gameplay in older adults with cognitive impairment. In this study, SGs were found to significantly increase cognitive ability and daily behavioral capacity and decrease depression.

Previous studies have explored the effects of digital SGs on cognitive ability [57], daily behavioral capacity [95], and depressive emotion problems [96] in patients with MCI or AD. However, previous studies have reported inconsistent findings regarding the effects of digital SG therapy on cognitive function [21,60-62], daily behavioral capacity [52,63], and depression [21,50,64-66] in older adults with MCI or AD. This systematic review included the recent literature (2017-2023) and examined 3 indicators of the effects of SGs on cognitive ability, daily behavioral capacity, and depression among older adults with MCI or AD. Consistent with previous findings, our results indicated positive effects of SGs on MMSE [97] and CSDD [50,98] scores and negative effects on depression on EDG-15, EADG, and GDS scores. In addition, there was a significant increase in cognitive ability (measured with MoCA) and daily behavioral capacity (measured with the ADL scale, the K-MBI, the Instrumental Activities of Daily Living (IADL) scale, the Katz Index, the Barthel Index, and the Lawton and Brody Index). This systematic review is the first to simultaneously test the effects of SGs on the 3 indicators of cognitive ability, daily behavioral capacity, and depression. This systematic review also divided the subgroup analysis of weeks and continental plates, which was not available in previous systematic reviews.

SGs are defined as games that are not for entertainment purposes and are mainly used for prevention, education, diagnosis, screening, and rehabilitation [99,100]. The word first appeared in 1968 when Abt [101] named his new book on SGs. According to Clark’s [101] proposed treatment, games can exist in a variety of forms, including (1) exergames or video games that require physical exercise as part of playing the games [38,39]; (2) cognitive training games, which aim to maintain or improve users’ cognitive abilities (eg, executive function, memory, learning) [34-37]; (3) computerized CBT games, which are video games that provide CBT to the users; and (4) biofeedback games, which are video games that use electrical sensors attached to the player to receive information about the player’s body state (eg, electrocardiogram sensors) and seek to influence some of the player’s body functions (eg, heart rate). Special care units (SCUs), also known as dementia care units, began to appear in the United States in the 1980s for older adults with dementia [102]. With an increase in the number of older adults with dementia and the diversification of the demand for dementia care in the market, these SCUs provide not only services to older adults in institutions but also family counseling, care support, and community therapy services to older adults with MCI [103]. At the policy level, the United States also launched the National Plan to Address Alzheimer’s Disease in 2012, with the goal of preventing and effectively treating AD by 2025 [104]. The latest SG therapy has also had a positive impact on easing the stress of dementia among older adults in US society [66]. However, the attention to older patients with MCI and AD in the West is much earlier than in China. Model-based projection data put China’s older adults with dementia at 6.73-15.7 million in 2025; the number of older adults with dementia will increase to 10.018-23.3754 million in 2035 [105]. China’s Circular on the Promotion of Dementia Prevention and Treatment Action (2023-2025) [106] formally focuses on groups such as older adults with MCI and AD at the national policy level. Ren et al [11] found that the 2015 annual treatment cost of patients with AD in China was US $167.74 billion, with ever-rising treatment costs expected to reach US $1.8 trillion by 2050. Older care facilities, social work stations, and counseling services in China may use SGs to carry out nonpharmacological treatments for patients with MCI and AD. This will provide both physical and psychological health improvements to older adults and at the same time reduce the financial burden on China. In traditional Chinese society, positive family and social relationships contribute to physical and mental health [107]. Due to the “Xiao Dao” culture, this also constitutes a unique social security system for older adults [108,109]. However, rapid industrialization and urbanization have changed these traditional arrangements [110]. Today, older adults are less likely to live with and interact with younger generations, which has resulted in older adults being isolated [111]. This has a direct impact on access to social care and financial security and even affects the physical and mental health of older adults [112]. SGs can provide opportunities for older adults to interact with others [113] and reduce the negative emotional impact on them [114]. In addition, the sports elements included in SGs [115] can exercise older adults’ cognitive ability [67] and daily behavioral capacity [116] and may even promote intergenerational relationships [117]. Our findings also show that SGs can have a positive impact on cognitive ability and daily behavioral capacity and decrease depression in older adults. With this in mind, we suggest that nursing home caregivers, social workers, and psychologists consider using SGs in their work and collaborate with SG professionals to bring benefits to older adults with MCI or AD. This will slow down the cognitive loss in older adults with MCI or AD to a certain extent.

### Strengths and Limitations

Compared with previous studies [90,118,119], this review is the only one to specifically evaluate the effects of SGs on older adults and related types of games. Because the study was conducted in strict accordance with PRISMA guidelines, it was a high-quality, transparent review. Only RCTs were included; this is the most rigorous method of studying causal relationships [120,121]. Therefore, the conclusions of this review are more credible. In this review, there was minimal publication bias. We searched 12 worldwide databases and included research from the United Kingdom, China, Belgium, the Netherlands, Portugal, Spain, and Korea. We also performed forward and backward checks of reference lists, and an all-around search strategy was applied. We searched a database of gray literature without restricting the search to a specific comparator, population, setting, or country, which made the samples more representative. Since all the processes (ie, data extraction, research selection, evidence quality assessment, and bias risk assessment) were independently conducted by 4 reviewers, there was no risk of bias in this review. In addition, because we used the Grading of Recommendations Assessment, Development, and Evaluation (GRADE) method to evaluate the quality of evidence, this review will enable readers to draw more accurate conclusions. Where possible, our statistics integrate data, which will increase not only the power of the research but also estimates of the effects of digital SG therapy on cognitive ability, daily behavioral capacity, and depression. Moreover, due to the large sample size, we performed a subgroup analysis and a sensitivity analysis. In addition, this review used pre- and postintervention outcome data from each group to evaluate the effectiveness of each meta-analysis. All previous studies have reported the SD and average overall cognitive changes pre- and postintervention in each group. In addition, in these studies, there were significant differences in overall cognition between groups at baseline.

The limitations of this study should also be considered. First, due to the design of the systematic review, a causal relationship with a clear structure could not be inferred. Second, the outcome indicators of anxiety (EADG scores) and quality of life (Dementia Quality of Life (DQoL)) were also related to the mental health of older adults with cognitive impairment. However, with only 1 study reporting these findings, a comparison could not be made, so we did not include these indicators. Third, because of the limitations of the designs of the included studies, the randomization and blinding methods used were seldom described in detail. Only 11 studies described the randomization method. In contrast, randomization was only mentioned in other studies, but no explanation of the method used was provided. Blinding was only implemented in 7 studies. Fourth, since many potentially eligible studies were pilot RCTs or quasiexperiments published before 2017 or in languages other than English, we excluded them. As a result, some related research may have been missed. The reason for excluding these studies is that the internal validity of pilot RCTs and quasiexperiments is lower than that of RCTs [122,123]. In addition, we were also unable to translate all the non-English research literature. In our previous review, we found some studies published before 2017. However, serious progress has been made in the field of gaming in the past 7 years, so we did not include RCTs published before 2017. Fifth, this review focused on the short-term effects of SGs. This meta-analysis of pre- and postintervention data did not include follow-up data. We found that follow-up data were reported in only 4 studies, and there was no consistency between the follow-up periods. Therefore, it was impossible to evaluate the long-term effects of SGs on the cognitive ability, mental health, and daily behavioral capacity of older adults. The quality of the evidence in our meta-analysis was average, which may have affected the internal validity of the findings. Sixth, in this review, interest intervention was limited to SGs used as therapeutic interventions on digital platforms, so it would not have been possible to comment on and check the effectiveness of SGs and nondigital SGs used for other purposes, such as monitoring, screening, or diagnosis.

### Research and Practical Implications

Since the review concentrated on the effectiveness of SGs in improving cognitive ability, daily behavioral capacity, and depression among older adults with cognitive impairment, researchers should evaluate the effectiveness of SGs by performing further reviews of their positive effects on cognitive abilities (eg, processing speed, executive function, learning, memory, speed), daily behavioral capacity (eg, executive function, capacity for reaction, upper limb activity), and mental health (eg, anxiety, stress, sleep quality, quality of life) among people in different age groups. We included some studies from middle-income countries, so the generalizability of the findings of this review may be limited to these countries. As the cultural, social, and economic conditions of middle-income countries are different, additional relevant research should be carried out. To date, only a few studies have assessed the effectiveness of SGs in improving cognitive ability. Therefore, additional research is needed to help draw clearer conclusions about the effectiveness of SGs. Due to the lack of follow-up data, the long-term effects of SGs were not evaluated in this review. To assess the long-term cognitive effects of SGs in a worldwide context, researchers should follow up with participants. A few studies did not report the SD or average global cognitive changes in each group before and after the intervention. In the future, relevant studies need to report this information to evaluate the effect more accurately. Because most of the existing studies have problems reporting results and choosing the randomization process, 8 studies had a low risk of overall bias. Therefore, when developing and reporting on RCTs, researchers should use recommended tools (eg, RoB-2 [124]) and follow recommended guidelines to avoid the aforementioned biases. Many studies have examined the worldwide impact of digital SGs on the cognition of healthy older adults [57], but this review revealed that only 14 studies have investigated the effects of digital SGs on older adults with cognitive impairment. We hope that researchers can perform additional research in this area to fill this gap.

According to the results of this review, SGs, especially digital SGs, exhibit greater effectiveness than a lack of interventions. These findings have meaningful implications for clinical practice since the use of SGs can improve cognitive ability and daily behavioral capacity and decrease depression in older adults with cognitive impairment. However, these findings should be interpreted with caution because the quality of the evidence is low in most meta-analyses. Moreover, most of the studies included in this review had some problems in terms of overall bias. Except for 7 studies [34,39,41-43,45,47], in all the other included studies, heterogeneity was high and the total effect was not accurate enough. Therefore, before more strong evidence is obtained, psychiatrists and psychologists should consider SGs as a complement to existing interventions rather than a direct substitute. In addition, the number of older persons worldwide will grow exponentially in the next few years, so we can try to use SGs to lighten the burden on the health care system. We can use SGs to improve the physical, psychological, social, and motor functions of older persons with cognitive impairment, thereby improving their quality of life [54,125]. Currently, many old people live alone and have limited social interactions, which can often lead to increased morbidity and mortality [126]. Older adults can play SGs in a relatively cozy environment (ie, at home), thus promoting their connections with friends and family [51]. In all the studies, the platforms for SGs were mobile devices (tablets and smartphones). Mobile devices are more popular than game consoles, cheaper than computers, and easier to access; thus, they are particularly popular. As of 2021, there were more than 7.1 billion mobile device users and 15 billion mobile devices worldwide [127]. Game and app developers can work together to develop SGs aimed at improving the mental health, daily behavior capacity, and cognitive ability of older people. These SGs can be operated and played using mobile devices. A survey of a small number of studies conducted in middle-income countries revealed that these countries seem to prefer SGs more than high-income countries do [128]. Middle-income countries lack many psychological professionals (currently 1 for every 10 million people). Therefore, more SGs should be developed in middle-income countries to improve the cognitive ability, mental health, and daily behavioral capacity of older persons in those countries.

### Conclusion

Overall, SGs, particularly digital SGs, may be associated with increased cognitive function and daily behavioral capacity, as well as decreased depression, among older adults with cognitive impairment. However, we are still unable to draw definitive conclusions on the safety and effectiveness of SGs in improving the cognition, behavioral capacity, and mental health of older people with cognitive impairment. This is due to the poor quality of existing meta-analysis evidence, which is mainly caused by concerns about the bias of most studies, the accuracy of the total effect, and the high degree of evidence heterogeneity. Therefore, until more reliable evidence is obtained, psychiatrists, psychologists, patients, and social workers should all regard SGs as a complement to existing interventions rather than a direct substitute. Before SGs are used, their effectiveness and safety should be further reviewed and evaluated, mainly in terms of improving daily behavioral capacity (eg, reaction ability, executive function, upper limb activity) of people with or without cognitive impairment in different age groups, cognitive ability (eg, processing speed, executive function, learning, memory), and mental health aspects (eg, stress, anxiety, sleep quality, quality of life). Further research is needed to assess the safety of SGs, the effects of sports games, and their long-term effects. In the future, further research on methods for increasing the motivation of participants, reducing the rate of missing data, and maintaining the effects of SGs is needed. Second, due to study limitations, it was impossible to perform subgroup analyses for intervention types. Consequently, in-depth and stratified discussions and comparisons of different intervention types were performed. In the coming years, it will be necessary for researchers to perform higher-quality studies with larger samples to verify the effectiveness of SG for older adults with cognitive impairment.

## Appendix

Multimedia Appendix 1 Preferred Reporting Items for Systematic Reviews and Meta-Analyses (PRISMA) 2020 abstract checklist.

Multimedia Appendix 2 Preferred Reporting Items for Systematic Reviews and Meta-Analyses (PRISMA) 2020 checklist.

Multimedia Appendix 3 Search strategy.

Multimedia Appendix 4 Intervention characteristics.

## Abbreviations

AD: Alzheimer’s disease
ADL: activities of daily living
CBT: cognitive behavioral therapy
CG: control group
CSDD: Cornell Scale for Depression in Dementia
DQoL: Dementia Quality of Life
EADG: Goldberg Anxiety and Depression Scale
EDG-15: 15-item Geriatric Depression Scale
GDS: Geriatric Depression Scale
IADL: Instrumental Activities of Daily Living
K-MBI: Korean version of the Modified Barthel Index
MCI: mild cognitive impairment
MMSE: Mini-Mental State Examination
MoCA: Montreal Cognitive Assessment
PICOS: Population, Intervention, Comparison, Outcomes, and Study Design
PRISMA: Preferred Reporting Items for Systematic Reviews and Meta-Analyses
RCT: randomized controlled trial
SCU: special care unit
SG: serious game
SGG: severe game group
SMD: standardized mean difference

## Data Availability

In this study, the original contributions have been included in the supplementary materials, and further inquiries can be made directly from the corresponding author. No artificial intelligence was used in any portion of the manuscript writing.

All the claims made by this paper represent only those of the authors and do not necessarily represent the claims of the affiliated organizations or reviewers, editors, and publishers. The publisher does not guarantee or endorse any product that may be evaluated by this paper or any claim that may be made by its manufacturer.
