# Correction: Effects of Electronic Serious Games on Older Adults With Alzheimer’s Disease and Mild Cognitive Impairment: Systematic Review With Meta-Analysis of Randomized Controlled Trials

**DOI:** 10.2196/65184

**Published:** 2024-08-14

**Authors:** Xinyi Zuo, Yong Tang, Yifang Chen, Zhimiao Zhou

**Affiliations:** 1 Sociology Department School of Government Shenzhen University Shenzhen, Guangdong China; 2 Institution of Policy Studies Lingnan University Tuen Mun, Hong Kong SAR China (Hong Kong); 3 Shenzhen Senior High School Shenzhen China

In “Effects of Electronic Serious Games on Older Adults With Alzheimer’s Disease and Mild Cognitive Impairment: Systematic Review With Meta-Analysis of Randomized Controlled Trials” (JMIR Serious Games 2024;12:e55785) the authors noted two errors.

In the “Search Strategy” section, the dates used for keyword search were changed from:

The keywords were used to search for papers published from January 1, 2017, to December 1, 2023.

To the following:

The keywords were used to search for papers published from January 1, 2017, to December 31, 2023.

Additionally, in the “Data Extraction” section, the following information appeared:

The first author (XZ) used the modified version of the data extraction table in the Systematic Review Manual of Cochrane Interventions to extract data.

This has been changed to read as:

The co-first author (XZ) used the modified version of the data extraction table in the Systematic Review Manual of Cochrane Interventions to extract data.

The correction will appear in the online version of the paper on the JMIR Publications website on August 14, 2024, together with the publication of this correction notice. Because this was made after submission to PubMed, PubMed Central, and other full-text repositories, the corrected article has also been resubmitted to those repositories.

