# Supplementary material for: Effects of Electronic Serious Games on Older Adults With Alzheimer’s Disease and Mild Cognitive Impairment: Systematic Review With Meta-Analysis of Randomized Controlled Trials
Source: JMIR Serious Games. 2024 Jul 31;12:e55785. doi: 10.2196/55785 (PMC11324188; doi:10.2196/55785)
Supplement: Multimedia Appendix 3 [file games_v12i1e55785_app3.docx]

**Multimedia Appendix 3 Search strategy**

PubMed:

1. "Cognitive Ability"[Mesh] OR ("Daily Behavior Ability" OR " Mental Health" OR "Depression")[Title/Abstract]

2. "Serious Games Based"[Mesh] OR " Serious Games"[Mesh] OR "Web-Based or Mobile"[Mesh]

3. ("Old Adults in Cognitive Impairment" OR "Old Adults" OR "Older People")[Title/Abstract]

4. "A pilot study" OR "Randomized Controlled Trial" OR "RCT"[Title/Abstract]

5. 1 and 2 and 3 and 4

6. Filters: Humans, Chinese, English

Embase:

1. ("Cognitive Ability" OR "Daily Behavior Ability" OR "Mental Health" OR "Depression"):ab,ti

2. ("Serious Games Based" OR " Serious Games" OR "Web-Based or Mobile":ab,ti)

3. ("Old Adults in Cognitive Impairment" OR " Old Adults”" OR "Older People":ab,ti)

4. ("A pilot study" OR "Randomized Controlled Trial" OR "RCT":ab,ti)

5. 1 and 2 and 3 and 4

Wed of Science:

1. topic: ("Cognitive Ability" or"Daily Behavior Ability" OR "Mental Health" OR "Depression")

2. topic: ("Serious Games Based" OR "Serious Games"OR "Web-Based or Mobile")

3.topic: (“Old Adults in Cognitive Impairment ” OR “Old Adults” OR “Older People”)

4.topic: (“A pilot study” OR “Randomized Controlled Trial” OR “RCT”)

5. 1 and 2 and 3 and 4

Scopus:

1. ("Cognitive Ability" OR "Daily Behavior Ability" OR "Mental Health" OR "Depression") AND (LIMIT-TO (LANGUAGE, "English") OR LIMIT-TO (LANGUAGE , "Chinese" ) )

2. ("Serious Games Based" OR "Serious Games" OR "Web-Based or Mobile")

3. ("Old Adults in Cognitive Impairment" OR "Old Adults" OR "Older People")

4. ("A pilot study" OR "Randomized Controlled Trial" OR "RCT")

1 AND 2 AND 3 AND 4

Cochrane Library:

MeSH descriptor: [Cognitive Ability] explode all trees

2. MeSH descriptor: [Daily Behavior Abilit] explode all trees

3. MeSH descriptor: Mental Health] explode all trees

4. MeSH descriptor: [Depression] explode all trees

5. 1 or 2 or 3 or 4

6. MeSH descriptor: [Serious Games Based] explode all trees

7. MeSH descriptor: [Serious Games] explode all trees

8. MeSH descriptor: [Web-Based or Mobile] explode all trees

9. 6 or 7 or 8

10. MeSH descriptor: [Old Adults in Cognitive Impairment ] explode all trees

11. MeSH descriptor: [Old Adults] explode all trees

12. MeSH descriptor: [Older People] explode all trees

13. 10 or 11 or 12

14. MeSH descriptor: [A pilot study] explode all trees

15. MeSH descriptor: [Randomized Controlled Trial] explode all trees

16. MeSH descriptor: [RCT] explode all trees

17. 14 or 15 or 16
