# Supplementary material for: Effects of Electronic Serious Games on Older Adults With Alzheimer’s Disease and Mild Cognitive Impairment: Systematic Review With Meta-Analysis of Randomized Controlled Trials
Source: JMIR Serious Games. 2024 Jul 31;12:e55785. doi: 10.2196/55785 (PMC11324188; doi:10.2196/55785)
Supplement: Multimedia Appendix 4 [file games_v12i1e55785_app4.docx]

**Table S1.** Intervention characteristics (adapted from the template for intervention description and replication [TIDieR] checklist and guide).

| **Author, year** | **Intervention type** | | **Design (how)—the modes of delivery** | **Content (what)—the materials, procedures, activities, and/or processes** | **Delivery (who, where, when, how much)—format of the intervention delivery, the**  **location, duration of intervention, length of sessions, frequency of**  **sessions, intensity** | **Measurement** | **Follow-up** | **Jadad score** |
| --- | --- | --- | --- | --- | --- | --- | --- | --- |
|  | SGG^[[1]](#footnote-0)^ | CG^[[2]](#footnote-1)^ |  |  |  |  |  |  |
| 34 Yang, et al. (2017) | Brain-Care | The control group performed the usual care | Brain-Care : a cognitive enhancement software that improves the brain function, which distinguishes the ability to significantly lower each cognitive area, focusing on the functions of the seven cognitive domains, thereby improving the brain cognitive ability of the domain and enhancing the brain’s higher level functions. | Each question was provided according to the guidance of voice and subtitles, and the subject provided a solution  within a predetermined time by touching the screen with his  finger, or moving the finger while touching. In addition, after  each step, the results were automatically stored and designed  to make the patient feel fulfilled. | Format: Individual Location: hospital  Duration: 4 weeks Length: 60 min Frequency:2 times/week Intensity: The Seoul Neuropsychological Screening Battery was used  for the neuropsychological assessment of each cognitive function area of the patient. | K-MMSE^[[3]](#footnote-2)^, GDS^[[4]](#footnote-3)^ | after the intervention | 4 |
| 35 Kwan, et al. (2020) | Brisk Walking Intervention | the conventional behavior | Samsung Galaxy smartphone J2 with 2 apps (i.e., Samsung Health and WhatsApp) | Participants set weekly goals of brisk walking. Participants wear a step‐counter during week. Participants receive WhatsApp weekly routine messages, messages when there is no brisk walking for more than 2 days, and praise message when the weekly goal is achieved earlier than expected | Format: Individual Location: Anywhere the participant walks Duration: 12 weeks Length: 60 min Frequency: 7 times/week Intensity: Based on baseline fitness and progress | MoCA | Mid-intervention, Post intervention | 5 |
| 36 Lim, et al. (2023) | a serious game: Brain Talk™ | no training | Brain Talk™, a serious game for the elderly with MCI, consists of 20 cognitive and sensory training programs. | Brain Talk™ consists of 20 cognitive and sensory training programs. Cognitive area training involves memory, attention, language, visual fields, mathematics, planning, and thinking. The sensory area is related to coordination,  agility, and audiovisual responses. Participants could perform cognitive training with a tablet regardless of the time and place. | Format: Individual Location: homes  Duration: 4 weeks Length: 30 min Frequency: 3 times/week Intensity: Twelve levels of difficulty were adjusted  according to cognitive ability | K-MMSE, K-MoCA | after the intervention | 4 |
| 37 Lee, et al. (2018) | the new cognitive rehabilitation program | The COMCOG, the 1st clinically licensed device in Korea | a new computerized cognitive rehabilitation program (Better Cognition: Bettercog, M3 solution, Daegu, Korea) using touchscreen-based interface | The computerized cognitive program consisted of orientation (time, place, person), attention, memory, language, executive function, visuospatial function, calculation, motor functions and game. | Format: Individual  Location: In a separate space  Duration: 3 weeks  Length: 30 min  Frequency: 4 times/week  Intensity: 400 words based on literatures and cognitive training tools | K-MMSE, ADL | after the intervention | 4 |
| 38 Yang Fengzhen, (2021) | somatosensory interactive games | routine nursing intervention | Feel touch provided by Beijing intelligent catharsis "Brain training", "fruit ninja" and "badminton" three individual sense of interactive games | Patients keep standing, feet distance and shoulder width, treatment guide patients with Bobath handshake, the body feeling remote control grip, according to the game instructions, move the remote control, cut fruit, playing while say fruit name, "badminton", play in the front left foot, right foot, center of gravity in the right foot, according to the prompt serve, receive, ball, waving remote control, and left foot alternately, and calculated by the patient each score. | Format: Individual Location: hospital Duration: 12 weeks Length: 60 min Frequency: 5 times/week Intensity: based on cognitive functions, including orientation, memory, attention and computation, recall, and language ability, are positively assigned | MMSE^[[5]](#footnote-4)^、ADL^[[6]](#footnote-5)^ | after the intervention | 6 |
| 39 Van Santen, et al. (2020) | Exergaming group | the regular activity program but were not offered exergaming | interactive cycling using a stationary bicycle connected to a screen | They can pick a route, and it mimics the experience of cycling outside, thus offering simultaneous physical and cognitive stimulation. | Format: Group Location: community center/ welfare house  Duration: 8 weeks Length: 60 min Frequency: 5 times/week Intensity: mobility disability in older adults and consists of 3 subtests: balance, gait speed, and chair stands | MMSE | Mid-intervention, Postintervention | 5 |
|  |  |  |  |  |  |  |  |  |
| 40 Zheng, et al. (2018) | Microsoft Ki-ect 2.0 motion sensing interactive device, XBOX360 game console | routine nursing intervention | Microsoft Ki-ect 2.0 motion-sensing interactive device, XBOX360 game console, LED display. The game device breaks through the past simple to handle the key input operation | The "Fruit Ninja" game project was chosen for the intervention in this study, Play through changes in body movements | Format: Group Location: community center/ welfare house  Duration: 8 weeks Length: 60 min Frequency: 5 times/week Intensity: Through self-comparison and comparison with others, patients are encouraged to actively participate in the game and gain a sense of self-accomplishment. | MMSE, ADL, CSDD^[[7]](#footnote-6)^ | after the intervention | 6 |
| 41 Wu, et al. (2020) | trained with sensory stimulation | The control group performed the usual care | music intracavitary /Touch therapy/Body-sensing interactive game training | ①Guide the patient to keep the feet apart with the shoulders to ensure the body balance when the gravity is shifting; ② Guide the knee flexion of the virtual person to speed up the track; ③ When the gravity can change the direction of the virtual character to pass smoothly, the flag gate of the same color on the track; ④ guides the patient to change the knee joint from flexion position to extension position, and the virtual character can jump over the cliff to shorten the completion time. Then score patient with MoCA | Format: Individual Location: hospital  Duration: 12 weeks Length: 30 min Frequency: no data Intensity:) based on individual cognitive function & Wandering behavior monitoring | MoCA^[[8]](#footnote-7)^ | after the intervention | 4 |
| 42 Savulich, et al. (2017) | The Cambridge Neuropsychological Test Automated Battery Paired Associates Learning | The control group performed the usual care | Using a touch-sensitive  computer screen, boxes are displayed and opened in a randomized  order. | It is used to assess motor  speed and thus acts as a control measure of general alertness  to help interpret other cognitive tasks. An arrow will appear on  either the left or right side of a computer screen. After the arrow  appears, the participant is instructed to press a corresponding  left or right button, using a response box, as quickly as possible. | Format: group Location: hospital  Duration: 4 weeks Length: 60 min Frequency:2 times/week Intensity: participants rate  their experience in terms of enjoyment, desire to continue, level  of self-confidence, and self-rated memory ability using 10-cm  VAS. | MMSE | after the intervention | 5 |
| 43 Swinne, et al. (2021) | The exergame device | the usual care | The exergame device consisted of a pressure-sensitive step training platform on which participants performed stepping movements to play the games. | The starting position of each session was an upright stance with both feet in the middle of the platform. Participants interacted with the game interface by pushing one foot on one of the four different arrows. When the game required the player to perform a step to  the left or right, the associated lower limb was used. For a step in the two other directions, the player used a lower limb of preference. | Format: individual Location: long-term care facility  Duration: 8 weeks Length: 15 min Frequency:3times/week Intensity: automatically adapted the training level to the participants’ capabilities | MMSE, MoCA, CSDD, DQol^[[9]](#footnote-8)^ | after the intervention | 4 |
| 44 Liu, et al. (2022) | exergaming | maintain their usual daily physical activities. | The infrared light component of the Kinect system was used to capture and track changes in limb segment motion. | participants imitated a virtually-presented TC coach and responded to instant feedback by real-time adjustments in movement. The EXER-TC program includes changing standing from wide to narrow base, body mass weight shifting, squats, and slow symmetrical to diagonal coordination arm-leg movements | Format: individual Location: community Duration: 12 weeks Length: 50 min Frequency:3 times/week Intensity: those related to cognitive function, dual-task cost (DTC), and gait performance | MoCA | after the intervention | 6 |
| 45 Thapa, et al. (2020) | VR training | general health care | The VR training including instruction and eye stretching exercises, was performed with an Oculus VR headset(Oculus quest headset) and two wireless hand controllers. | Including: Juice making,Crow Shooting, Fireworks, Love house. | Format: Individual Location: care center Duration: 8 weeks Length: 100 min Frequency: 3 times/week Intensity: Based on Cognitive Function & Physical Function | MMSE | Mid-intervention, after intervention | 6 |
| 46 Oliveira, et al. (2021) | VR cognitive stimulation | as-usual care | The intervention was done using a computerized cognitive stimulation program with non-immersive VR. | The SLB comprised nine different tasks distributed in twelve sessions with different difficulty levels.Tasks T1–T6 were conducted inside a virtual apartment, whereas T7–T9 were outdoor tasks where participants needed to navigate to each of the locations in a virtual city. | Format: Individual Location: Residential care homes  Duration: 5 weeks Length: 45 min Frequency: 2 times/week Intensity: Using established neuropsychological instruments for assessing memory, attention, and executive functions. | MMSE, ADL | Mid-intervention | 2 |
| 47 Jahouh, et al. (2021) | Serious games | as-usual care, no VR | The intervention consisted of 20 rehabilitation sessions and made up of different activities with the Nintendo Wii Fit® video game console. | All sessions had four fundamental parts: (1) The games to be played in the session, as well as the necessary rules to execute them correctly, were explained to the participant by the therapist. (2) An aerobic-type game such as “Step” was used as a warm-up exercise. (3) The next game was played specifically to work on attention, concentration, and memory; this game is called “Nodding”. (4) To end the session, the participants had to choose a game that they wanted to try or play for a period of 5 min. | Format: Individual Location: nursing home Duration: 8 weeks Length: 40-45 min Frequency: 2-3 times/week Intensity: Using established | MMSE, ADL，EGD-15^[[10]](#footnote-9)^，EADG^[[11]](#footnote-10)^ | After the intervention | 6 |

1. a SGG, Serious game group. [↑](#footnote-ref-0)
2. b CG, control group. [↑](#footnote-ref-1)
3. g K-MMSE: Korean version of the Mini-Mental State Examination [↑](#footnote-ref-2)
4. h GDS: Geriatric Depression Scale. [↑](#footnote-ref-3)
5. c MMSE: Mini_Mental State Examination. [↑](#footnote-ref-4)
6. d ADL:Activities of daily living. [↑](#footnote-ref-5)
7. e CSDD:,Cornell Scale for Depression in Dementia. [↑](#footnote-ref-6)
8. f MoCA:Montréal Cognitive Assessment. [↑](#footnote-ref-7)
9. i DQol:Dementia Quality of Life [↑](#footnote-ref-8)
10. j EGD-15: Yesavage scale for Geriatric Depression. [↑](#footnote-ref-9)
11. k EADG: Goldberg Anxiety and Depression Scale. [↑](#footnote-ref-10)
